# CT-guided radiofrequency ablation of a tibial osteoid osteoma in pregnancy

**DOI:** 10.1007/s00256-026-05304-1

**Published:** 2026-07-23

**Authors:** Prisca C. Diala, Ilina Pluym, Nabia Khan, Joe Hong, Aisling Murphy, Varand Ghazikhanian, Simon Dadoun

**Affiliations:** 1 Department of Obstetrics and Gynecology, David Geffen School of Medicine, University of California, Los Angeles, USA; 2 Division of Maternal Fetal Medicine, Department of Obstetrics and Gynecology, David Geffen School of Medicine, University of California, Los Angeles, USA; 3 Department of Anesthesiology and Perioperative Medicine, David Geffen School of Medicine, University of California, Los Angeles, USA; 4 Department of Radiological Sciences, David Geffen School of Medicine, University of California, Los Angeles, USA

**Keywords:** Osteoid osteoma, Pregnancy, CT-guided radiofrequency ablation in pregnancy, Radiation safety in pregnancy

## Abstract

Osteoid osteomas are painful benign bone tumors that primarily affect the long bones and may be managed conservatively with nonsteroidal anti-inflammatory drugs (NSAIDs). When surgical management is required, minimally invasive techniques such as image-guided radiofrequency ablation (RFA) are preferred. Management of osteoid osteomas during pregnancy poses a clinical challenge as NSAIDs are often contraindicated and there are limited reports describing surgical treatment in pregnancy. We present the case of a 32-year-old woman at 34 weeks’ gestation who presented with severe right lower extremity pain exacerbated at night. CT imaging of the tibia revealed an 8 mm subperiosteal nidus with focal cortical thickening, periosteal bone formation, and a small central calcification consistent with an osteoid osteoma. The patient had minimal pain relief with acetaminophen and escalating opioid requirements and thus underwent an uncomplicated ultrasound and CT-guided RFA of the lesion with resolution of pain. The total CT dose length product was 81 mGy-cm, well within the acceptable radiation threshold of pregnancy, and minimized by use of ultrasound for localization and needle advancement. The patient subsequently had an uncomplicated term vaginal delivery. In pregnant patients for whom NSAIDs are not a viable treatment option for osteoid osteoma, this case demonstrates that image-guided RFA can be a safe and effective management strategy when performed by a multidisciplinary team comprising of musculoskeletal radiology, maternal-fetal medicine, and anesthesiology.

## Introduction

Osteoid osteoma is a benign bone tumor characterized by a small radiolucent nidus, typically less than 1.5 cm in diameter, which produces high levels of prostaglandins and results in significant pain [1, 2]. It is the third most common bone tumor, accounting for 12% of all benign bone tumors in young adults [3]. Lesions are usually solitary and most commonly involve the long bones [4, 5]. Patients classically present with progressively worsening nocturnal pain, and computed tomography (CT) is considered the gold standard for diagnosis [6].

Osteoid osteomas may resolve spontaneously or be treated conservatively with nonsteroidal anti-inflammatory drugs (NSAIDs), which inhibit prostaglandin production and provide effective pain control [7, 8]. However, this treatment is not effective in all patients, and there is a lengthy time interval (30–40 months) for complete symptomatic relief that may be poorly tolerated [8]. Surgical intervention is usually required for those with progressive or refractory pain or those with functional limitation. In these cases, image-guided ablation therapies including CT-guided radiofrequency ablation (RFA) have emerged as a safe, effective, and minimally invasive option [9, 10].

Management of osteoid osteoma in pregnancy presents unique challenges. NSAIDs are contraindicated in pregnancy because they inhibit cyclooxygenase-mediated prostaglandin synthesis, which is essential for maintaining fetal renal perfusion and patency of the ductus arteriosus [11]. In the second and third trimesters, NSAID exposure is associated with fetal renal dysfunction leading to oligohydramnios and with premature constriction or closure of the ductus arteriosus, resulting in fetal pulmonary hypertension [11]. Additionally, concerns regarding fetal radiation exposure may limit the use of CT-guided intervention in pregnant patients [12]. This case report demonstrates that CT-guided RFA can be safely and effectively performed during pregnancy with appropriate multidisciplinary planning and fetal radiation mitigation strategies. These findings have important clinical relevance for the management of pregnant patients with severe, refractory pain secondary to an osteoid osteoma.

## Case report

A 32-year-old G2P1001 at 34 weeks’ gestation presented to Labor and Delivery (L&D) with a 7-month history of progressively worsening right lower extremity pain that began early in pregnancy, was exacerbated at night, and limited weight bearing. The patient’s prior workup was notable for an MRI lumbar spine with no localizing lesions, and they were advised to defer CT imaging until the postpartum period. The patient had trialed a course of steroids and physical therapy and was currently on a regimen of acetaminophen 1000 mg every 6 h and gabapentin 300 mg three times daily with minimal relief.

**Fig. 1:**
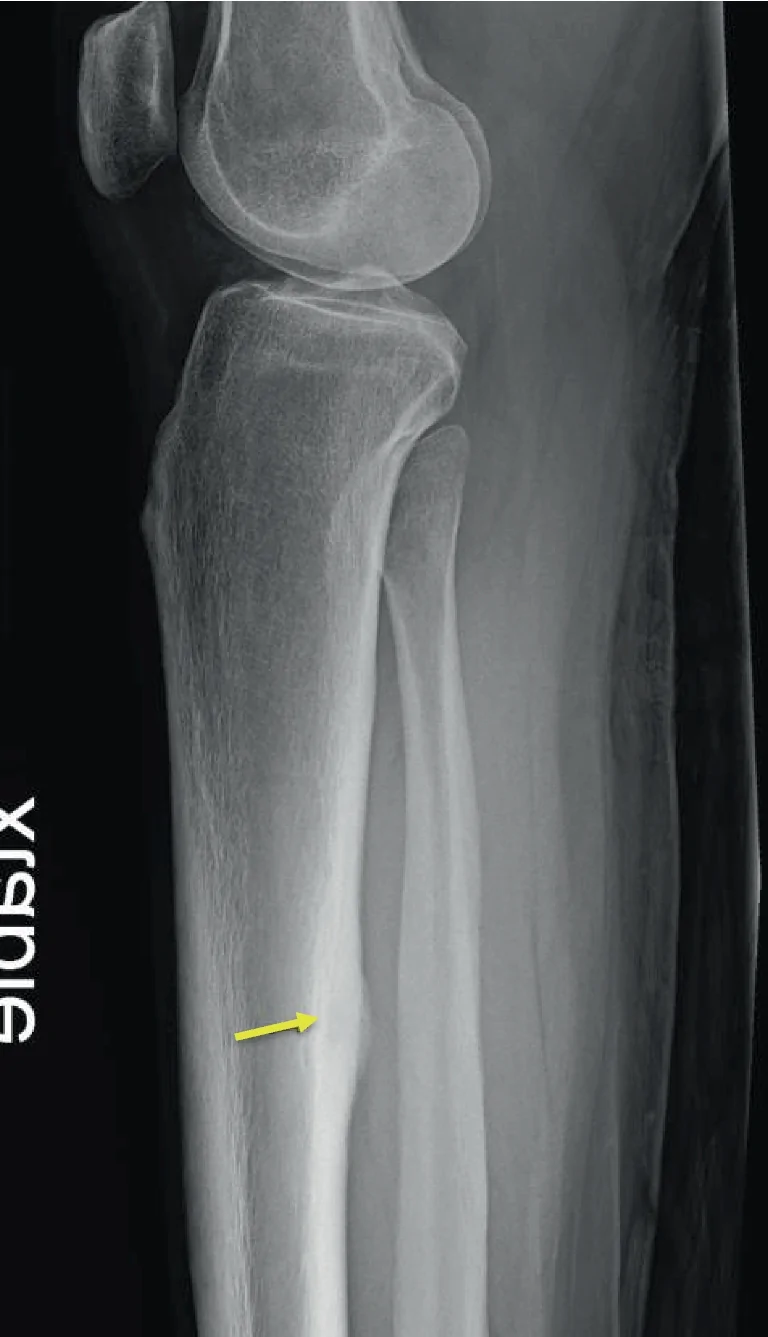
Lateral radiograph of the tibia and fibula with focal cortical thickening and periosteal bone formation of the posterior right tibial proximal diaphysis

**Fig. 2:**
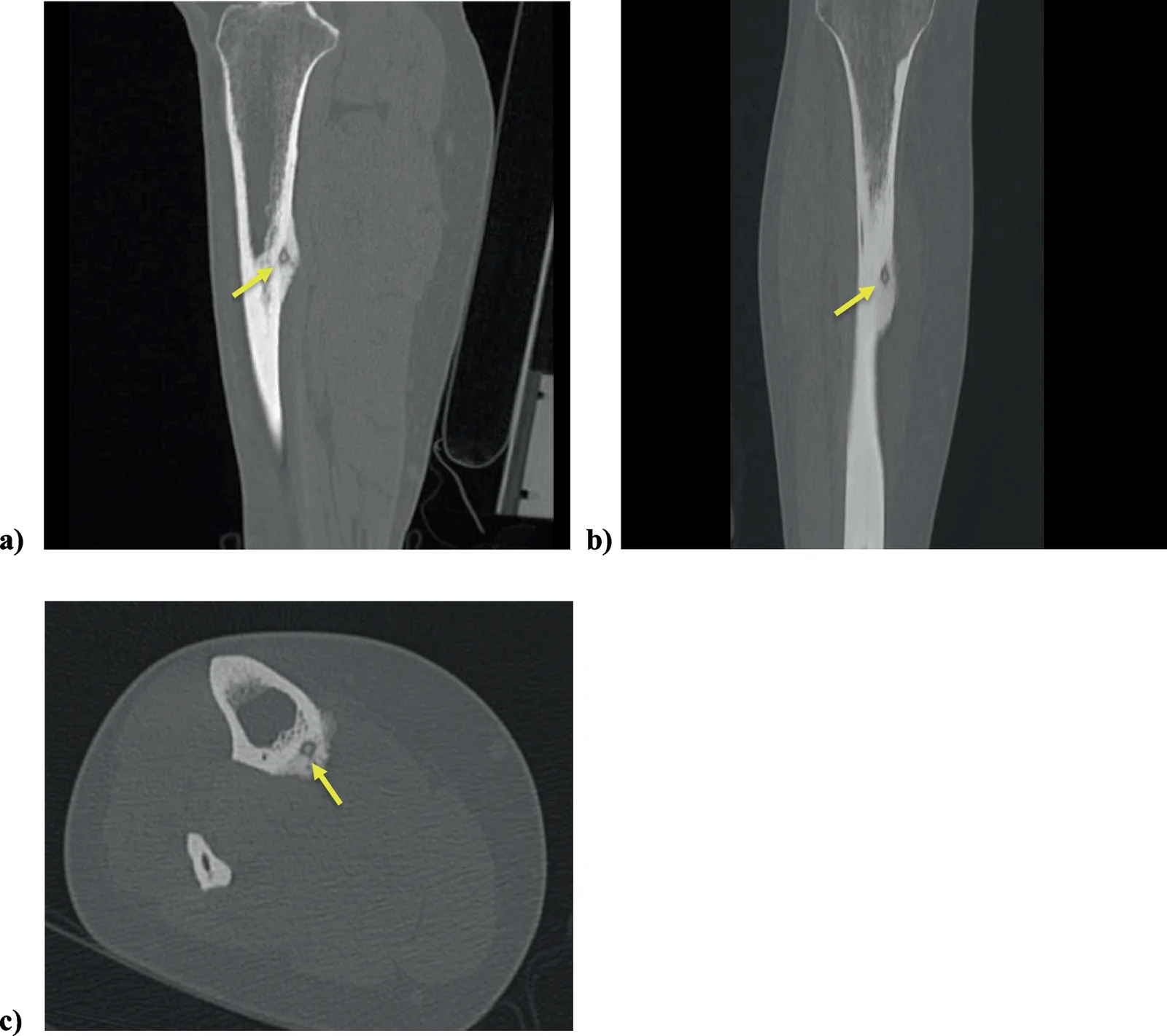
Multiple CT images of the right tibia and fibula demonstrating a subperiosteal nidus along the posterior medial mid tibial diaphysis with central calcifications and associated cortical thickening and periosteal bone formation in **a** sagittal, **b** coronal, and **c** axial views

Radiographs of the tibia and fibula demonstrated a focal area of cortical thickening and periosteal bone formation along the medial and posterior aspect of the mid tibia, suspicious for an osteoid osteoma (Fig. 1). CT of the tibia and fibula confirmed an 8 mm subperiosteal nidus with focal cortical thickening, periosteal bone formation, and a small central calcification within the posterior medial aspect of the mid tibial diaphysis, consistent with an osteoid osteoma (Fig. 2). Despite inpatient management with a hydromorphone patient-controlled analgesia infusion, pain remained intractable. Musculoskeletal radiology was thus consulted for evaluation and consideration for percutaneous CT-guided RFA, and Anesthesiology was consulted for intraoperative and postoperative pain control management.

To minimize maternal sedation, a saphenous nerve block was attempted but did not provide sufficient analgesia. A recommendation was then made to proceed with a combined spinal-epidural (CSE) anesthetic. At 35 weeks’ gestation, the patient underwent percutaneous CT- and ultrasound-guided biopsy and RFA in the interventional radiology (IR) suite under a CSE. Fetal heart rate was monitored continuously in the Interventional Radiology suite by an L&D nurse and the maternal-fetal medicine team. The patient was positioned with left lateral tilt, a Foley catheter was placed, and the patient’s abdomen and neck were covered with a lead apron to protect against ionizing radiation.

**Fig. 3:**
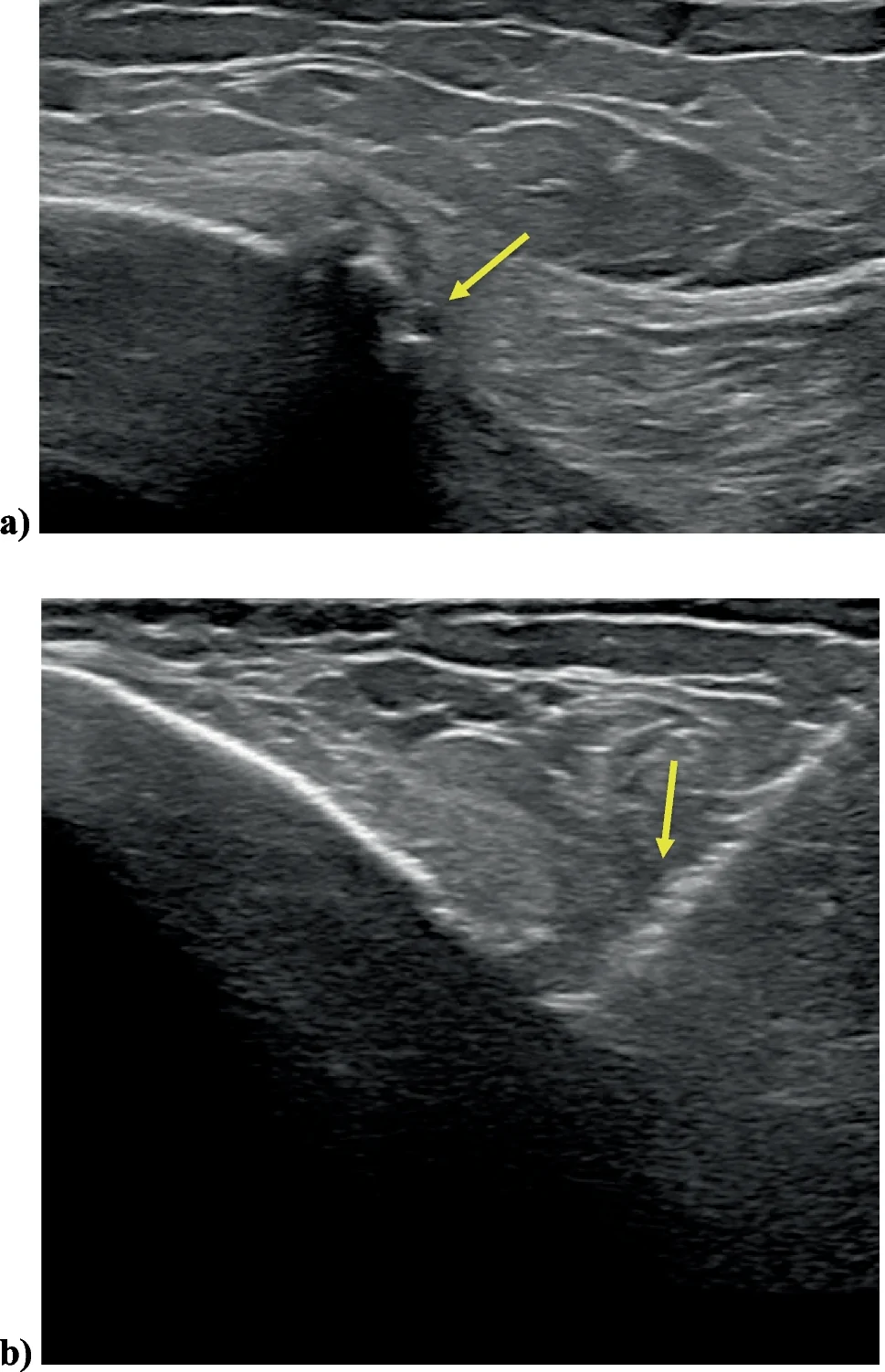
Ultrasound guided **a** localization of lesion and **b** needle advancement

**Fig. 4:**
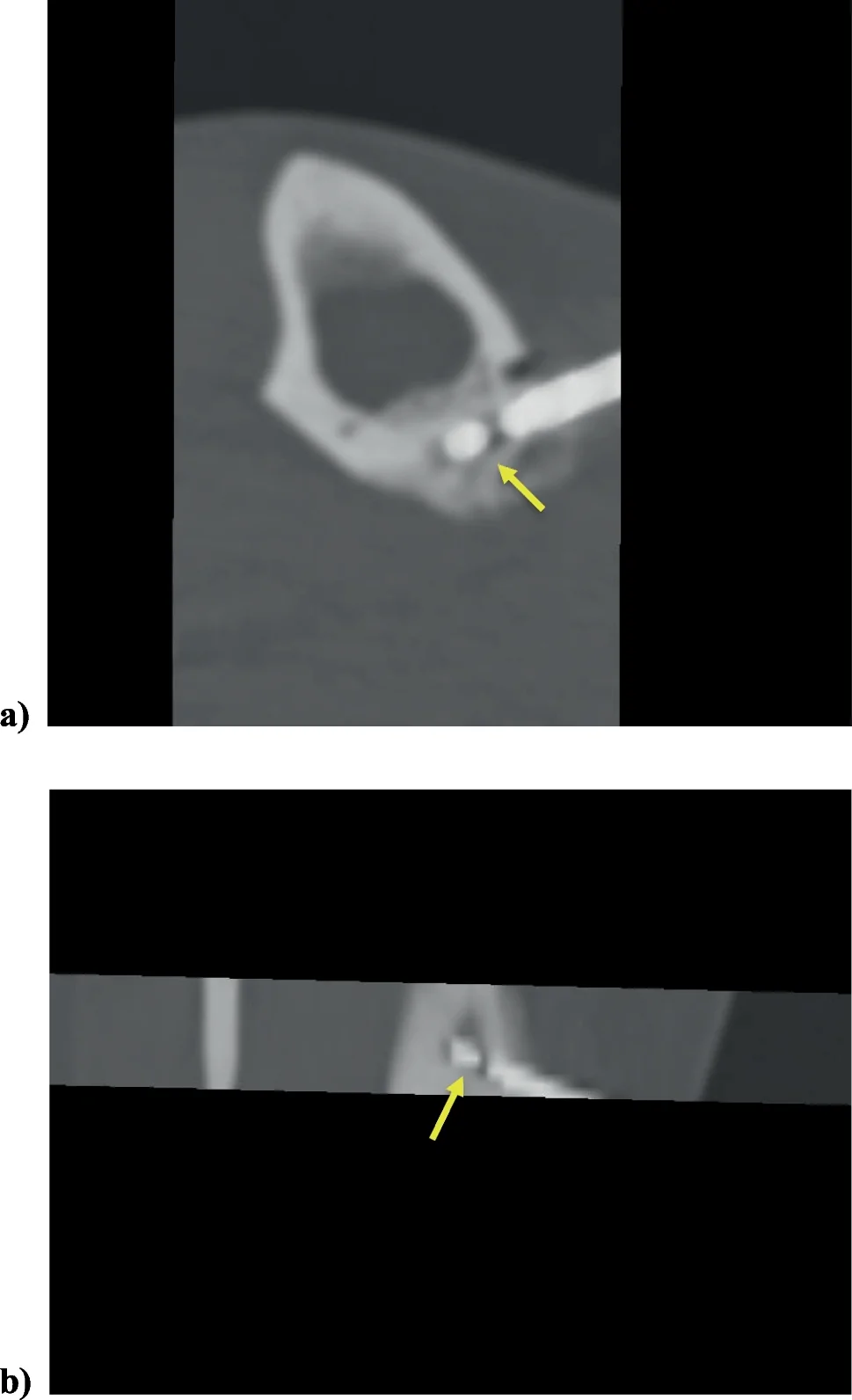
CT-guided RFA of the subperiosteal nidus in **a** axial and **b** coronal oblique views. Arrows identify the radiofrequency ablation probe tip within the nidus, which is partially obscured by the probe

Under combined spinal-epidural (CSE) anesthesia, a 9 × 5 × 4 mm area along the right posterior medial tibial diaphysis was localized. The patient was prepped and draped in a sterile fashion, and a percutaneous core biopsy specimen was obtained using a Becton Dickinson Trek device with a 10-gauge introducer and a 12-gauge needle. A Stryker OptaBlate probe was then advanced into the nidus under combined ultrasound and CT guidance (Figs. 3 and 4). Ablation was performed at 90 °C for 6 min. Post-procedural CT imaging confirmed appropriate probe positioning without hematoma, fracture, or other complications. Estimated blood loss was less than 10 mL, and total CT dose length product was 81 mGy-cm. Fetal heart rate monitoring for 4 h post-procedure was reassuring.

The epidural component of the CSE allowed for adequate pain control postoperatively, and the patient’s pain was additionally managed with oral acetaminophen 1000 mg every 8 h and oxycodone 5 mg every 4 h as needed. By postoperative day one, the patient reported significantly improved pain, and by 1 week the patient was pain free. The pathology report of the core biopsy confirmed an osteoid osteoma. The remainder of the patient’s pregnancy was uncomplicated, and they subsequently delivered a healthy term neonate via vaginal delivery. The patient remained asymptomatic at their 6-week postpartum visit.

## Discussion

CT-guided RFA is well established as a safe, effective, and minimally invasive treatment option for osteoid osteoma [9, 10, 13]. However, its use in pregnancy has rarely been described. To the best of our knowledge, there is only one prior reported case involving a 23-year-old patient who underwent CT-guided RFA of a distal femoral osteoid osteoma at 28 weeks’ gestation [14]. The limited number of documented cases is possibly due to concerns regarding fetal radiation exposure in pregnancy. This case demonstrates that when deferring treatment to the postpartum period is not feasible due to functionally disabling and refractory pain, CT-guided ablation can be safely performed in pregnancy with appropriate planning.

Pregnancy limits the use of NSAIDs due to increased risk of oligohydramnios and premature closure of the ductus arteriosus after 32 weeks’ gestation [11] and often necessitates escalation of opioid analgesia. Prolonged opioid use in pregnancy carries both maternal risk of dependency and fetal risk of developing neonatal abstinence syndrome [15]. Given this, definitive management with CT-guided RFA may provide meaningful maternal benefit while reducing prolonged opioid exposure.

Concerns regarding ionizing radiation exposures in pregnancy are related to potential fetal risks of lethality, congenital anomalies, fetal growth restriction, and intellectual impairment [16, 17]. However, these risks depend on both the gestational age at the time of exposure as well as the dose of radiation exposure. Risk of fetal lethality is highest during the preimplantation period, structural malformations are most likely during organogenesis (weeks 2–8), and neurodevelopmental impairment becomes the principal concern beyond approximately 25 weeks of gestation [17]. A minimal threshold for these adverse effects has been suggested to range from 60 to 310 mGy, but the lowest clinically documented dose associated with severe intellectual disability is 610 mGy [17–19]. In this case, the patient was treated at 35 weeks’ gestation, which reduced the risk of structural malformations and minimized the risk of neurodevelopmental impairment compared with treatment at earlier gestational ages. The CT dose length product (DLP) of 81 mGy-cm from the CT-guided RFA procedure (KV:80, mAs: 80, CTDIvol: 1.31–1.67 mGy), which was limited to the tibia, was within recognized fetal safety thresholds. Although the total DLP of 746 mGy-cm from the diagnostic CT of the tibia and fibula exceeds this threshold, imaging was confined to the lower extremity and abdominal lead shielding was used, thereby resulting in negligible fetal exposure to ionizing radiation.

Although this patient’s lesion was distal, spinal osteoid osteomas are less frequent, accounting for approximately 10% of cases, and may present greater technical and feasibility challenges [20]. These challenges include safe positioning during pregnancy and access to spinal lesions as gestational age advances. As the gravid uterus enlarges and extends beyond the pelvis, it increasingly involves the lumbar and lower thoracic regions, creating additional challenges for both radiation minimization during CT of the abdomen and pelvis and positioning for procedural or surgical management. However, if the pregnant patient can be safely positioned and access to these spinal regions can be obtained, radiography, CT imaging, and CT-guided RFA may be performed during pregnancy when clinically indicated and after shared decision-making [21]. These procedures should be paired with radiation mitigation strategies such as minimizing scan range, optimizing radiation dosage, shielding, and maximizing procedural efficiency. A similar risk-benefit framework would be applied at earlier gestational ages, with individualized considerations of fetal risk, symptom severity, and available alternatives.

Available alternative treatment options for osteoid osteoma during pregnancy include MR-guided focused ultrasound (MRgFUS), percutaneous cryoablation, and surgical excision [22, 23]. Compared with CT-guided RFA, MRgFUS is a noninvasive approach that avoids ionizing radiation exposure, has demonstrated comparable clinical response [24, 25], and may be a useful alternative at earlier gestational ages, when concern for radiation-related structural malformations is greater. However, its availability is limited, and treatment may be technically challenging depending on lesion location, which may preclude adequate positioning. Percutaneous cryoablation, which can be performed under CT or MRI guidance, has been established as a safe and effective long-term option for pain control [23]. MRI-guided cryoablation may be particularly appealing in pregnancy because it eliminates ionizing radiation while allowing real-time visualization of the ice ball and adjacent structures. Surgical excision also avoids ionizing radiation but is more invasive, typically requires general anesthesia, and has been associated with longer recovery times and a greater risk of structural weakening or fracture compared with percutaneous ablation techniques, which may increase maternal morbidity during pregnancy [26]. Therefore, minimally invasive approaches are generally preferred in pregnancy. However, choice of treatment modality is often institution and provider dependent.

With regard to anesthetic planning, no currently available anesthetic agent demonstrates teratogenic effects in humans at standard concentrations at any gestational age [18]. However, anesthetic management during pregnancy must balance adequate maternal analgesia with fetal safety and readiness for obstetric emergencies. In this case, a saphenous nerve blockade was initially chosen to minimize adverse effects on maternal-fetal physiology. However, it failed to provide adequate analgesia, likely due to the lesion’s deep subperiosteal location. General anesthesia was avoided, given pregnancy-associated airway risks and greater systemic fetal exposure. A CSE was therefore selected to provide dense procedural analgesia. This approach also limited systemic opioids and sedatives, reducing the risk of maternal respiratory depression and fetal acid-base disturbances. At the same time, the epidural component provided the opportunity to extend the blockade for the procedure, readiness for emergent obstetric intervention, and to provide postoperative analgesia. To help maintain adequate placental perfusion and mitigate the hypotension associated with spinal anesthesia, aggressive intravenous resuscitation was given in the perioperative period.

From a technical standpoint, ultrasound was used to identify the lesion, thereby avoiding the need for initial CT localization imaging and allowing ultrasound-guided advancement of the needle to the target area, which reduced the number of CT scans required. Short-segment CT images were subsequently obtained to confirm and adjust needle position within the nidus.

Given the gestational age and location of the lesion in the proximal to mid tibial diaphysis, concern regarding overall anesthesia duration was considered greater than concern regarding fetal radiation exposure in this case. Accordingly, CT- and ultrasound-guided RFA was determined by the multidisciplinary team to be the most reasonable and accessible treatment option. If the lesion had been more centrally located and anesthesia duration were less limiting relative to radiation exposure, MR-guided cryoablation would have been preferred; however, this modality was not available at our institution and would likely have required a longer procedure time even if available. Likewise, the peripheral lesion location and positioning constraints would have made treatment with MRgFUS technically challenging and likely prolonged the procedure.

In future cases, during pregnancy, all three modalities may be considered, with final selection guided by lesion location, resource availability, and multidisciplinary decision-making. This case adds to the limited evidence supporting percutaneous CT-guided RFA of osteoid osteomas during pregnancy and suggests that pregnancy alone should not be an absolute contraindication when symptoms are severe and refractory. The decision to proceed must remain individualized, balancing gestational age, pain severity, prior medical therapy, and availability of a multidisciplinary team.

## Data Availability

Not applicable.
